# Prognostic Value of Inflammatory and Metabolic Biomarkers in ICU-Admitted Trauma Patients: A Retrospective Cohort Study

**DOI:** 10.3390/medicina61091530

**Published:** 2025-08-26

**Authors:** Hasan Celik, Basak Pehlivan, Veli Fahri Pehlivan, Erdogan Duran

**Affiliations:** 1 Department of Anesthesia and Reanimation, Çermik State Hospital, Ministry of Health, 21100 Diyarbakır, Türkiye; hcelik101010@gmail.com; 2 Department of Anesthesia and Reanimation, Faculty of Medicine, Osmanbey Campus, Harran University, 63300 Sanliurfa, Türkiye; bpehlivan@harran.edu.tr (B.P.); dreduran@harran.edu.tr (E.D.)

**Keywords:** trauma, intensive care unit, mortality prediction, prognostic biomarkers, Platelet-to-Lymphocyte Ratio (PLR), C-reactive Protein-to-Albumin Ratio (CAR), lactate, base deficit

## Abstract

*Background and Objectives:* Prognostic stratification in trauma patients admitted to the intensive care unit (ICU) remains a clinical challenge. While conventional scoring systems such as Acute Physiology and Chronic Health Evaluation II (APACHE II), Injury Severity Score (ISS), and Glasgow Coma Scale (GCS) are widely used, the utility of biochemical biomarkers in predicting mortality is still evolving. This study aimed to evaluate the prognostic value of key inflammatory and metabolic biomarkers: platelet-to-lymphocyte ratio (PLR), C-reactive protein-to-albumin ratio (CAR), serum lactate, base deficit, and neutrophil-to-lymphocyte ratio (NLR) in relation to ICU mortality in trauma patients. *Materials and Methods:* In this retrospective cohort study, data from 240 ICU-admitted trauma patients were analyzed. Group comparisons between survivors and non-survivors were conducted using t-tests or Mann–Whitney-U tests. Pearson correlation and ROC analyses were performed to assess relationships and discriminatory performance of biomarkers alongside clinical scores. *Results:* Non-survivors (*n* = 50) exhibited significantly higher CAR, lactate, and base-deficit values, and lower PLR (*p* < 0.05) compared to survivors (*n* = 190). CAR strongly correlated with CRP (r = 0.96), while lactate and base deficit were inversely correlated (r = –0.69). ROC analysis revealed that ISS (AUC = 0.86) and APACHE II (AUC = 0.77) had the highest discriminatory power, followed by lactate (AUC = 0.75). NLR did not demonstrate significant prognostic utility (*p* > 0.05). *Conclusion:* PLR, CAR, lactate, and base deficit are accessible, cost-effective biomarkers with significant prognostic value in ICU trauma care. Their integration with established scoring systems can enhance early risk stratification. NLR, however, may require time-sensitive and context-specific evaluation.

## 1. Introduction

Trauma remains a major global public health issue, and is one of the leading causes of morbidity and mortality worldwide. According to data from the World Health Organization (WHO), trauma-related deaths and disabilities account for a significant portion of the global burden of disease [1,2]. Traumatic injuries, particularly prevalent among the young population, not only adversely affect individual quality of life, but also impose a substantial burden on healthcare systems [3]. Severe trauma cases often require multidisciplinary management and close monitoring in intensive care units (ICUs). In this patient population, accurate and early prognostication is critical for optimizing treatment strategies and reducing mortality rates [4,5,6,7].

In the management of trauma patients in intensive care units, there is growing interest in the use of objective and reliable biomarkers alongside traditional clinical assessments. These biomarkers provide valuable insights into patient prognosis by reflecting the severity of the inflammatory response, the extent of tissue injury, and the risk of organ dysfunction [8]. Over the past 15 years, research has demonstrated that the systemic inflammatory response and immune dysfunction following trauma can resemble a sepsis-like state, with various hematological and biochemical parameters showing prognostic significance in this context [9].

In this regard, inflammation-associated markers such as the neutrophil-to-lymphocyte ratio (NLR), platelet-to-lymphocyte ratio (PLR), and C-reactive protein-to-albumin ratio (CAR), as well as markers of tissue perfusion such as serum lactate and base deficit, have emerged as potential tools for predicting mortality and morbidity in trauma patients. NLR, a simple yet effective indicator of systemic inflammation and physiological stress, reflects the imbalance between elevated neutrophils and decreased lymphocytes [4]. Elevated NLR values have been associated with poor prognosis and increased mortality risk in trauma patients, particularly those with traumatic brain injury (TBI) [10,11]. PLR, due to the role of platelets in hemostasis and their involvement in inflammatory pathways, has been suggested as a prognostic marker in acute inflammatory and prothrombotic states [12]. Lower PLR values have been shown to correlate with increased mortality in patients with severe trauma [13,14]. CAR, an index reflecting both the degree of inflammation and nutritional status, has been linked to adverse clinical outcomes in critically ill patients [15]. Elevated CAR levels have been highlighted as an independent risk factor for predicting mortality in polytrauma patients [15,16]. Furthermore, lactate and base-deficit levels obtained from arterial blood-gas analysis serve as early indicators of tissue hypoperfusion and metabolic acidosis, and have been strongly correlated with increased mortality in trauma populations [17,18].

Given the need for rapid and reliable risk assessment in critically injured patients, inflammation-based biomarkers and perfusion-related biochemical parameters are increasingly being investigated for their prognostic relevance in trauma care. While scoring systems such as the Injury Severity Score (ISS), Glasgow Coma Scale (GCS), and APACHE II remain widely used, simple and cost-effective biomarkers like the neutrophil-to-lymphocyte ratio (NLR), platelet-to-lymphocyte ratio (PLR), C-reactive protein-to-albumin ratio (CAR), serum lactate, and base deficit may offer additional insights into patient prognosis. However, the comparative prognostic value of these parameters in ICU-admitted trauma patients remains to be clarified. We hypothesized that routine biochemical markers (NLR, PLR, CAR, lactate, and base deficit), in combination with clinical scoring systems, could significantly predict ICU mortality in trauma patients.

The primary outcome of the study is to assess the association between these biomarkers and mortality. Secondary outcomes include the evaluation of NLR as a prognostic marker, comparison of the predictive performance of these biomarkers with established scoring systems such as the Injury Severity Score (ISS), Glasgow Coma Scale (GCS), and APACHE II, and the exploration of how trauma etiology and anatomical injury distribution relate to biomarker profiles. Through this analysis, we aim to provide clinically applicable insights that may enhance decision-making and prognostic accuracy in the early management of critically injured patients.

## 2. Materials and Methods

**Figure 1:**
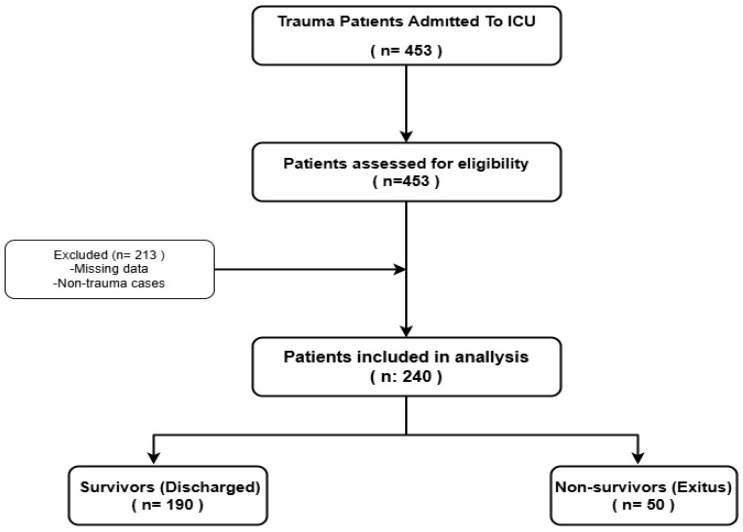
Flowchart of patient selection and group allocation.

This retrospective cohort study was conducted after obtaining approval from the Harran University Faculty of Medicine Clinical Research Ethics Committee (approval number: HRÜ/24.15.45). The study protocol was registered at ClinicalTrials.gov (Identifier: NCT06667310). Following written institutional permission from the hospital administration, patient data were extracted from the electronic medical records of the general intensive care unit (ICU) for the period between 1 January 2021, and 31 December 2023. This retrospective cohort study was conducted in accordance with the STROBE (Strengthening the Reporting of Observational Studies in Epidemiology) guidelines, ensuring comprehensive and transparent reporting of observational research (Figure 1).

Patients admitted to the ICU due to trauma including traffic accidents, falls (including those in the elderly), gunshot wounds, stab wounds, electrical injuries, assault-related trauma, and crush injuries (e.g., due to earthquakes) were screened. A total of 453 trauma patients were identified. Patients were included regardless of age or sex, provided that their treatment was completed and complete medical data were available. Patients who were still under treatment at the time of data collection or who had incomplete laboratory or clinical records were excluded. Ultimately, 240 eligible trauma patients were included in the analysis (Figure 1).

For each patient, the following data were extracted and anonymized: age, sex, length of ICU stay, trauma type, anatomical region of injury (head–neck, thorax, abdomen, extremities), comorbidities, Glasgow Coma Scale (GCS) score, Acute Physiology and Chronic Health Evaluation II (APACHE II) score, and Injury Severity Score (ISS). Laboratory parameters obtained within the first 24 h of ICU admission were recorded, including neutrophil, lymphocyte, platelet, C-reactive protein (CRP), albumin, lactate, and base-deficit values.

From these values, the following biomarker ratios were calculated:

- Neutrophil-to-lymphocyte ratio (NLR).
- Platelet-to-lymphocyte ratio (PLR).
- CRP-to-albumin ratio (CAR).

All hematological and biochemical measurements were conducted in the central hospital laboratory using standard automated analyzers. Arterial blood-gas analysis was used to determine lactate and base-deficit levels.

Patients were stratified into two outcome groups: survivors (discharged from ICU) and non-survivors (in-ICU mortality). Demographic, clinical, and laboratory characteristics were compared between these groups, to identify parameters associated with mortality. The primary study outcome was in-ICU mortality, while ICU length of stay was considered a secondary outcome. All eligible patients meeting the inclusion criteria during the specified period were enrolled, and no a priori sample-size calculation was performed. However, a post hoc power analysis based on ROC curve results was conducted to evaluate the adequacy of the sample size for key variables with the highest discriminative performance.

All biomarkers were measured on admission, within the first 24 h of ICU stay, to assess their immediate prognostic utility. Demographic variables, trauma characteristics, and laboratory results were extracted from electronic medical records. Operative status, American Society of Anesthesiologists (ASA) physical status classification, and butyrylcholinesterase (BChE) levels were not consistently available in the dataset, and were therefore not included in the analysis.

The neutrophil-to-lymphocyte ratio (NLR) was included, due to its frequent use in the literature as a readily available and cost-effective inflammatory biomarker in critically ill populations, including trauma. Other inflammatory and metabolic biomarkers (PLR, CAR, lactate, and base deficit) were selected, based on their established relevance in previous prognostic studies of ICU patients.

### Statistical Analysis

All analyses were performed using IBM SPSS Statistics version 27.0 (IBM Corp., Armonk, NY, USA). The normality of continuous variables was assessed using the Kolmogorov–Smirnov and Shapiro–Wilk tests. Normally distributed variables were presented as mean ± SD and compared using the independent samples t-test; non-normally distributed variables were expressed as median (IQR) and compared using the Mann–Whitney U test. Categorical variables were analyzed using the chi-square or Fisher’s exact test. A *p*-value < 0.05 was considered statistically significant.

Pearson correlation analysis was conducted to evaluate linear relationships among key biomarkers (NLR, PLR, CAR, lactate, base excess) and clinical severity scores (GCS, APACHE II, ISS). ROC curve analysis was used to determine the predictive performance of these variables for ICU mortality, and the area under the curve (AUC) was reported for each.

A post hoc power analysis was performed for variables with the highest AUC values, using the Hanley and McNeil method, to estimate the standard error (SE) and corresponding z-scores. For ISS (AUC = 0.86; n_1_ = 50 non-survivors, n_2_ = 190 survivors), SE was 0.0346 (z = 10.41), and for lactate (AUC = 0.75), SE was 0.0426 (z = 5.87). Both variables demonstrated a statistical power > 99% at a 95% confidence level, confirming that the available sample size was sufficient to detect significant discriminatory performance.

Additionally, for prospective study planning, an a priori sample-size calculation was conducted, assuming α = 0.05, 80% power, and a survivor:non-survivor ratio of approximately 3.8:1. The minimum required total sample size was estimated as N = 27 for ISS (AUC = 0.86) and N = 85 for lactate (AUC = 0.75), whereas parameters with lower AUCs such as CAR (AUC = 0.61) and CRP (AUC = 0.58) would require substantially larger samples (N > 500) to achieve adequate power.

## 3. Results

This retrospective cohort study analyzed the data of 240 trauma patients admitted to the general intensive care unit (ICU) of Harran University Medical Faculty between 2021 and 2023. From a total of 453 trauma admissions, only patients with complete medical records and completed treatment processes were included in the final analysis. The demographic characteristics, trauma etiology, injury sites, comorbidities, prognostic scoring systems, and biomarker levels were compared between survivors and non-survivors (Figure 1).

### 3.1. Demographic and Clinical Characteristics

Among the 240 patients included, 162 (67.5%) were male and 78 (32.5%) were female. The overall mean age was 34.02 ± 26.72 years (median: 26.50; range: 0–94). The total ICU mortality rate was 20.8% (*n* = 50). No statistically significant differences were observed between the survivor and non-survivor groups in terms of sex distribution (79.6% vs. 66% male, *p* > 0.05) or mean age (33.27 ± 26.88 vs. 36.88 ± 26.15 years, *p* > 0.05). However, the mean ICU length of stay was significantly longer in non-survivors (8.88 ± 11.78 days) compared to survivors (4.95 ± 5.82 days, *p* < 0.001), suggesting an association between prolonged ICU stay and poor prognosis (Table 1).

### 3.2. Prognostic Scoring Systems

Prognostic scoring systems (ISS, GCS, APACHE II) were found to be significantly associated with mortality (Table 2):

- Injury Severity Score (ISS): the mean ISS was significantly higher in non-survivors (20.76 ± 7.96) compared to survivors (10.12 ± 4.33, *p* < 0.001), indicating a strong association between injury severity and mortality risk.
- Glasgow Coma Scale (GCS): survivors had significantly higher GCS scores (13.23 ± 2.74) than non-survivors (5.68 ± 3.70, *p* < 0.001), confirming that low GCS is a strong predictor of mortality.
- APACHE II Score: the mean APACHE II score was significantly higher in non-survivors (22.96 ± 5.89) compared to survivors (10.98 ± 4.46, *p* < 0.001), indicating its prognostic value in predicting ICU mortality.

### 3.3. Biomarker Levels

The prognostic value of various biomarkers measured at ICU admission was as follows (Table 3):

- Neutrophil-to-Lymphocyte Ratio (NLR): NLR values were slightly lower in non-survivors (7.95 ± 8.83) than in survivors (9.37 ± 8.09), but this difference was not statistically significant (*p* > 0.05).
- Platelet-to-Lymphocyte Ratio (PLR): PLR was significantly lower in non-survivors (126.16 ± 108.58) than in survivors (179.25 ± 124.12, *p* < 0.05), suggesting that reduced PLR is associated with increased mortality.
- CRP-to-Albumin Ratio (CAR): CAR was significantly higher in non-survivors (2.98 ± 5.75) than in survivors (0.73 ± 1.86, *p* < 0.05), confirming its predictive value for mortality.
- Serum Lactate: lactate levels were significantly elevated in non-survivors (4.99 ± 4.07) compared to survivors (2.39 ± 1.46, *p* < 0.05), indicating impaired tissue perfusion in the deceased group.
- Base Deficit: non-survivors showed a more profound base deficit (–7.24 ± 8.15) compared to survivors (–2.03 ± 4.33, *p* < 0.05), supporting its value as a marker of metabolic acidosis and poor outcome.

These findings suggest that PLR, CAR, serum lactate, and base deficit are valuable prognostic biomarkers for mortality in trauma patients, whereas NLR did not demonstrate statistically significant prognostic relevance in this study. The prognostic scoring systems (ISS, GCS, and APACHE II) were confirmed as robust tools for mortality risk stratification in critically injured trauma patients.

### 3.4. Performance Assessment of Prognostic Indicators in Trauma-ICU Patients Using Correlation and ROC Analyses

**Figure 2:**
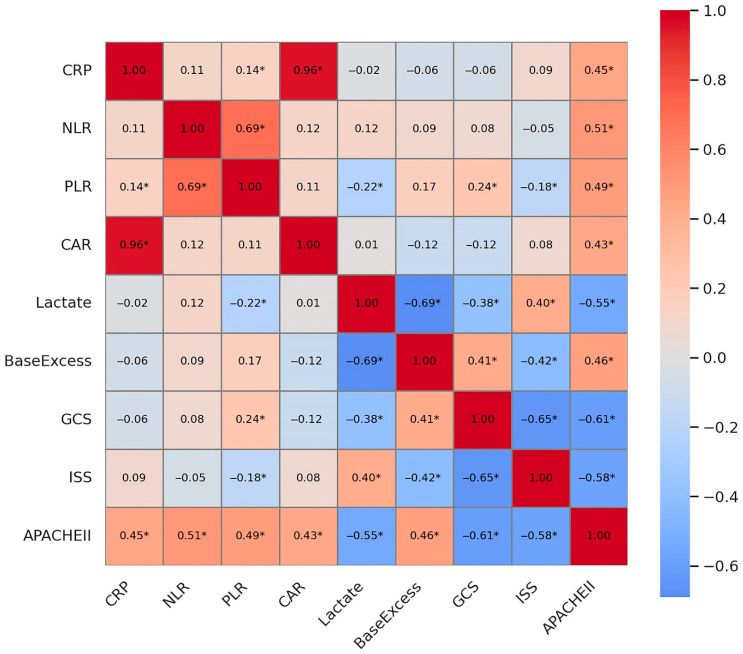
Pearson correlation matrix of prognostic markers and severity scores in ICU-Trauma patients. CRP, C-reactive protein; NLR, neutrophil-to-lymphocyte ratio; PLR, platelet-to-lymphocyte ratio; CAR, C-reactive protein-to-albumin ratio; GCS, Glasgow Coma Scale; APACHE II, Acute Physiology and Chronic Health Evaluation II; ISS, Injury Severity Score. * *p* < 0.05.

Figure 2 displays the Pearson correlation matrix among key prognostic biomarkers and clinical scoring systems, including CRP, NLR, PLR, CAR, lactate, base excess, GCS, APACHE II, and ISS. Statistically significant correlations (*p* < 0.05) are marked with an asterisk (*). Notably, CAR showed a strong positive correlation with CRP (r = 0.96, *p* < 0.001), indicating that the CRP/albumin ratio is heavily driven by CRP levels. Lactate and base excess were significantly inversely correlated (r = −0.69, *p* < 0.001), reflecting the acid-base derangement in critically ill patients. GCS showed significant negative correlations with lactate, base excess, APACHE II, and ISS scores, and was inversely correlated with mortality, underscoring its value as a clinical prognostic indicator. These findings support the interrelated nature of biochemical stress markers and clinical severity scores in predicting trauma outcomes.

**Figure 3:**
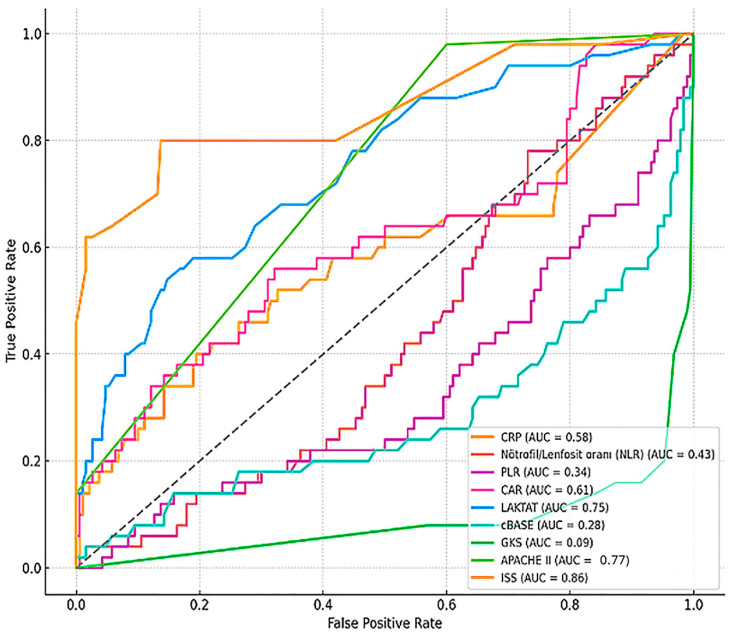
ROC curve comparison of biomarkers and clinical scores for mortality prediction. ROC, receiver operating characteristic; AUC, area under the curve; ISS, Injury Severity Score; APACHE II, Acute Physiology and Chronic Health Evaluation II. The dashed line indicates the reference line (AUC = 0.5).

Figure 3 illustrates the receiver operating characteristic (ROC) curves of individual biomarkers and clinical scores for predicting in-hospital mortality. Among the evaluated parameters, the ISS (AUC = 0.86) and APACHE II (AUC = 0.77) demonstrated the highest discriminative ability. Lactate (AUC = 0.75) and CAR (AUC = 0.61) also showed moderate predictive performance, while CRP and NLR had poor discriminatory value (AUC = 0.58 and 0.43, respectively). These results suggest that while inflammatory markers offer limited standalone prognostic value, composite severity scores and lactate levels remain more reliable indicators of mortality risk in trauma patients admitted to intensive care.

## 4. Discussion

This retrospective cohort study aimed to comprehensively evaluate the prognostic utility of readily accessible biomarkers, including neutrophil-to-lymphocyte ratio (NLR), platelet-to-lymphocyte ratio (PLR), C-reactive protein-to-albumin ratio (CAR), lactate, and base deficit, in predicting mortality among trauma patients admitted to the intensive care unit (ICU). Our findings largely align with the evolving understanding of trauma pathophysiology and prognostication over the past 15 years, while also presenting a nuanced perspective on the role of NLR that warrants further exploration.

The demographic profile of our cohort predominantly young males with high rates of falls and traffic accidents aligns with global and national trauma trends [2,3], likely reflecting occupational exposure, risk-taking behavior, and socioeconomic factors. Elevated mortality from firearm and thoracic injuries concurs with literature linking high-energy trauma and vital organ damage to poorer outcomes [19]. Additionally, the higher prevalence of comorbidities like hypertension among non-survivors highlights the role of chronic diseases in compromising physiological reserves and increasing vulnerability to complications, thereby worsening trauma prognosis [20].

Our results indicate that several established clinical scoring systems, namely ISS, GCS, and APACHE II, are robust predictors of ICU mortality in trauma patients [21,22]. Specifically, higher ISS and APACHE II scores, and lower GCS scores, were significantly associated with increased mortality. These findings are consistent with extensive literature findings, underscoring the critical role of comprehensive injury severity and physiological derangement assessments in trauma prognostication [21,22]. The strong discriminative power of ISS (AUC = 0.86) and APACHE II (AUC = 0.77) in our ROC analysis further reinforces their utility as primary tools for risk stratification.

Beyond traditional scores, our study highlights the significant prognostic value of several readily available biochemical markers. Elevated CAR, lactate, and base-deficit levels, along with reduced PLR, were significantly associated with increased ICU mortality. These findings are in agreement with recent studies emphasizing the role of systemic inflammation and tissue hypoperfusion in determining outcomes in critically ill trauma patients [6,13,15,16,17,18]. The strong positive correlation between CAR and CRP (r = 0.96) suggests that CAR primarily reflects the inflammatory burden, which is a key driver of adverse outcomes in trauma. Similarly, the inverse correlation between lactate and base deficit (r = −0.69) underscores their combined utility as indicators of metabolic acidosis and inadequate tissue oxygenation, both critical determinants of survival in severe trauma [15,23].

The combined interpretation of our correlation and ROC analyses provides a more comprehensive understanding of these markers. While individual biomarkers like lactate (AUC = 0.75) and CAR (AUC = 0.61) demonstrated moderate predictive performance, their utility is enhanced when considered alongside clinical scores [15,16,18]. For instance, the significant negative correlations of GCS with lactate, base deficit, APACHE II, and ISS scores highlight the interconnectedness of neurological status, metabolic derangement, and overall injury severity. This suggests that a multi-modal approach, integrating both clinical and biochemical parameters, offers superior prognostic accuracy compared to relying on single markers or scores alone [21,22].

The National Early Warning Score 2 (NEWS 2) is a widely adopted physiological scoring system designed to facilitate the early detection of clinical deterioration by monitoring six simple parameters: respiratory rate, oxygen saturation, systolic blood pressure, pulse rate, level of consciousness, and temperature [24]. Unlike APACHE II, which incorporates a broader range of laboratory and physiological variables to provide a comprehensive assessment of disease severity and mortality risk, NEWS 2 prioritizes rapid bedside applicability, requiring minimal laboratory input and allowing repeated assessments during the patient’s ICU or ward stay. In trauma settings, NEWS 2 may serve as a useful adjunct for early triage and ongoing monitoring, whereas APACHE II may offer superior prognostic discrimination for long-term outcomes [25]. A direct comparison of these scoring systems is provided in Table 4.

In contrast to some previous reports [4,5,10], NLR did not demonstrate significant prognostic utility in our cohort (AUC = 0.43, *p* > 0.05). This finding suggests that while NLR is a general marker of systemic inflammation, its predictive power in trauma may be highly context-dependent, influenced by factors such as the timing of measurement, specific trauma mechanisms, and patient comorbidities. Further research, potentially involving serial NLR measurements and larger, more diverse cohorts, is warranted to fully elucidate its role in trauma prognostication [5].

Regarding comorbidities, while our study observed a higher frequency of comorbidities in non-survivors (26%) compared to survivors (15.3%), statistical comparisons were not performed to confirm significant differences in comorbidity prevalence between the groups. This limitation should be considered when interpreting the impact of comorbidities on mortality in our cohort. Future studies should include robust statistical analyses, to assess the independent effect of specific comorbidities on trauma outcomes.

Our findings have several important clinical implications. The accessibility and cost-effectiveness of PLR, CAR, lactate, and base deficit make them valuable tools for early risk stratification in resource-limited settings. Their integration into existing clinical assessment protocols can provide clinicians with rapid insights into a patient’s physiological status and potential for adverse outcomes. For example, persistently elevated lactate and base deficit, even in the presence of seemingly stable vital signs, should prompt aggressive resuscitation and further investigation for occult hypoperfusion. Similarly, a rising CAR could signal an escalating inflammatory response requiring targeted interventions.

Furthermore, the strong predictive performance of ISS and APACHE II underscores the continued importance of these established scoring systems. While biomarkers offer complementary information, they should not replace comprehensive clinical evaluation. Instead, a synergistic approach, where biomarkers guide initial assessment and trigger more detailed investigations, can optimize patient management. For instance, a patient with a high ISS but relatively normal biomarkers might still require close monitoring due to anatomical injury, whereas a patient with a lower ISS but deranged biomarkers might be at higher risk for systemic complications.

Butyrylcholinesterase (BChE) is a liver-synthesized enzyme that has been proposed as a potential biomarker of systemic inflammation, metabolic stress, and adverse postoperative outcomes. Previous studies have reported that low BChE levels on the first and third postoperative days are associated with an increased risk of prolonged ICU stay and postoperative complications, although findings regarding its relationship with sepsis remain inconsistent [26]. In trauma patients, reduced BChE activity may reflect impaired hepatic synthetic capacity and heightened inflammatory burden, both of which could contribute to poor prognosis [26]. Although we did not measure BChE levels in our cohort, incorporating this biomarker into future prospective studies could help clarify its prognostic value for ICU mortality in trauma populations.

Limitations of our study include its retrospective design and the focus on admission biomarkers. Future prospective studies incorporating serial measurements of biomarkers, along with a broader range of clinical outcomes (e.g., 30-day mortality, long-term functional recovery), would provide a more complete picture [27]. Additionally, exploring the impact of specific trauma mechanisms and the presence of sepsis or other complications regarding biomarker profiles would enhance the generalizability of these findings.

## 5. Conclusions

In conclusion, our study reinforces the critical role of established clinical scoring systems and highlights the significant prognostic value of PLR, CAR, lactate, and base deficit in predicting ICU mortality in trauma patients. These accessible biomarkers, when interpreted in conjunction with clinical scores and considering their correlations, can enhance early risk stratification and guide timely interventions, ultimately contributing to improved patient outcomes in trauma care.

## Figures and Tables

**Table 1:** Demographic characteristics, trauma etiology, injury sites, and comorbidities by outcome groups.

Variable	Survivors (Mean ± SD or n [%])	Non-Survivors (Mean ± SD or n [%])	*t* Statistic/Chi-square	*p*
Age (years)	33.27 ± 26.88	36.88 ± 26.15	−0.85	0.26
Gender (Female)	61 (32.1%)	17 (34%)	−0.253	0.8
Gender (Male)	129 (67.9%)	33 (66%)		
ICU Stay (days)	4.95 ± 5.82	8.88 ± 11.78	−2.288	<0.001 *
Trauma Etiology				
Traffic Accident	70 (36.8%)	20 (40.0%)		
Falls	79 (41.6%)	12 (24.0%)		
Stab Injuries	4 (2.1%)	0 (0.0%)		
Gunshot Injuries	12 (6.3%)	9 (18.0%)		
Electric Shock	10 (5.3%)	2 (4.0%)		
Assault/Violence	7 (3.7%)	2 (4.0%)		
Crush Injuries (incl. Earthquake)	8 (4.2%)	5 (10.0%)		
Injury Region				
Head–Neck	115 (60.5%)	31 (62.0%)	−0.189	0.85
Thorax	40 (21.1%)	14 (28.0%)	−0.983	0.5
Abdomen	45 (23.7%)	14 (28.0%)	−0.606	0.53
Extremities	124 (65.3%)	30 (60.0%)	0.688	0.49
Comorbid Condition				
Hypertension	28 (14.7%)	9 (18%)		
Diabetes Mellitus	14 (7.4%)	6 (12%)		
Cardiovascular Disease	10 (5.3%)	3 (6%)		
Renal Failure	4 (2.1%)	2 (4%)		
Cerebrovascular Disease	3 (1.6%)	2 (4%)		
Asthma	2 (1%)	1 (2%)		
Epilepsy	2 (1%)	0 (0%)		

SD, standard deviation. Statistical analysis: independent samples *t*-test for continuous variables; chi-square or Fisher’s exact test for categorical variables. * *p* < 0.05.

**Table 2:** Association Between Prognostic Scoring Systems and Mortality in Trauma Patients.

Parameters	Survivors Mean ± SD (Score)	Survivors Median (Min–Max)	Non-Survivors Mean ± SD (Score)	Non-Survivors Median (Min–Max)	*t* Statistic	*p*
Glasgow Coma Scale	13.23 ± 2.74	15 (3–15)	5.68 ± 3.70	4 (3–15)	16.053	<0.001 *
APACHE II	10.98 ± 4.46	11 (1–24)	22.96 ± 5.89	23 (8–37)	−15.727	<0.001 *
ISS	10.12 ± 4.33	9 (2–22)	20.76 ± 7.96	20 (3–38)	−12.669	<0.001 *

ISS, Injury Severity Score; SD, standard deviation; Min, minimum; Max, maximum. Statistical analysis: independent samples *t*-test. * *p* < 0.05.

**Table 3:** Association Between Biochemical Parameters and Mortality in Trauma Patients.

Parameters	Discharged Mean ± SD	Discharged Median (Min–Max)	Deceased Mean ± SD	Deceased Median (Min–Max)	*t* Statistic	*p*
Neutrophils (10^3^/µL)	12.31 ± 6.47	11.3 (1.2–34.1)	15.18 ± 8.22	14.85 (1.5–36)	−2.624	0.023 *
Lymphocytes (10^3^/µL)	2.37 ± 2.02	1.6 (0.3–10.1)	3.59 ± 3.97	2.6 (0.2–25.5)	−3.031	0.004 *
Platelets (10^3^/µL)	267.37 ± 94.86	262 (69–628)	245.86 ± 100.49	243 (66–569)	1.409	0.16
Albumin (g/dL)	3.99 ± 0.64	4.1 (1.7–5.5)	3.21 ± 0.82	3.2 (1.5–4.9)	7.164	<0.001 *
C-Reactive Protein (mg/L)	2.44 ± 5.66	0.26 (0.01–47.4)	7.06 ± 11.88	0.7 (0.05–41.8)	−3.937	<0.001 *
NLR	9.37 ± 8.09	7.5 (0.3–54.7)	7.95 ± 8.83	5.65 (0.1–54.5)	1.086	0.133
PLR	179.25 ± 124.12	155.48 (22.46–697.5)	126.16 ± 108.58	86.64 (10.82–455)	2.759	0.017 *
CRP/Albumin Ratio	0.73 ± 1.86	0.06 (0–16.93)	2.98 ± 5.75	0.2 (0.01–27.33)	−4.588	<0.001 *
Lactate (mmol/L)	2.39 ± 1.46	1.9 (0.6–9.2)	4.99 ± 4.07	4.0 (0.8–19)	−7.250	<0.00 1*
Base Deficit **^†^** (mmol/L)	−2.03 ± 4.33	−1.9 (−18.7–7.7)	−7.24 ± 8.15	−6.05 (−31.4–9.1)	6.199	<0.001 *

SD, standard deviation; Min, minimum; Max, maximum; ^†^ negative values indicate base deficit. Statistical analysis: independent samples *t*-test or Mann–Whitney U test. * *p* < 0.05.

**Table 4:** Comparison of the National Early Warning Score 2 (NEWS 2) and APACHE II in trauma patients.

Feature	NEWS 2	APACHE II
**Purpose**	Early detection of clinical deterioration and need for escalation of care	Comprehensive assessment of disease severity and prediction of mortality risk
**Parameters assessed**	Six physiological parameters (RR, SpO_2_, SBP, pulse, consciousness, temperature)	Twelve physiological parameters + age + chronic health conditions
**Data requirements**	Bedside measurements only	Bedside + laboratory measurements
**Complexity**	Simple, quick to calculate, no specialized software	More complex, often requires software or scoring sheet
**Time to complete**	<2 min	5–10 min
**Frequency of use**	Repeated assessments during stay	Usually calculated once on admission (may be repeated if clinically indicated)
**Strengths**	Rapid, low-resource, applicable in pre-hospital and ED settings	Higher prognostic accuracy for ICU/hospital mortality
**Limitations**	Less accurate for long-term prognosis, not specific for diagnosis	Requires more data, less suitable for rapid triage
**Use in trauma**	Useful for initial triage and continuous monitoring	Better for comprehensive prognostic evaluation

Comparison of NEWS 2 and APACHE II in trauma patients. NEWS 2 enables rapid bedside assessment, whereas APACHE II offers more comprehensive prognostic evaluation.

## Data Availability

The datasets used and/or analyzed during the current study are available from the corresponding author on reasonable request.
